# Levels of Awareness Regarding Pediatric Eye Diseases Among Saudi Parents From the Hail and Al-Qassim Regions, Saudi Arabia

**DOI:** 10.7759/cureus.57871

**Published:** 2024-04-08

**Authors:** Layan K Alshammari, Lama A Alaradi, Afaf M Alanazi, Faisal F Almishali, Norah H Alabdullatif, Abrar Ali

**Affiliations:** 1 Department of Ophthalmology, King Khalid Hospital, Hail, SAU; 2 Department of Ophthalmology, University of Hail College of Medicine, Hail, SAU; 3 Department of Ophthalmology, King Fahad Specialist Hospital, Al-Qassim, SAU; 4 Medical School, College of Medicine, Qassim University, Al-Qassim, SAU

**Keywords:** saudi arabia., parents, attitude, practice, knowledge, prevalence, children, vision screening, eye problems, pediatric eye diseases

## Abstract

Background: The importance of children's eye health cannot be overstated as it has significant implications for personal life, education, career, health, financial status, and overall satisfaction. This study aims to assess the awareness of parents regarding pediatric eye diseases to identify potential gaps in knowledge. By understanding parental awareness, we can develop targeted educational initiatives to promote early detection, timely intervention, and overall improved eye health in children. This research seeks to contribute valuable insights for enhancing preventive measures and fostering healthier eyesight in younger generations.

Methodology: A descriptive cross-sectional study was conducted in the Hail and Al-Qassim regions, of Saudi Arabia. Parents with children aged less than 15 years were included. Targeted parents were selected consecutively using an online questionnaire for data collection. Data included parents' data, children's eye diseases, and parents' knowledge, practice, and attitude toward pediatric eye diseases.

Results: A total of 618 eligible parents were included, 429 (69.4%) from Al-Qassim and 189 (30.6%) from Hail. Parents' ages ranged from 20 to 59 years with a mean age of 35.4 ± 11.5 years. A total of 510 (82.5%) respondents were females. A total of 442 (71.5%) of the study parents had poor knowledge about their pediatric eye problems, 154 (24.9%) had a good knowledge level, and only 22 (3.6%) had excellent knowledge. As for their practice, 458 (74.1%) of the parents arranged for their children to undergo an eye or visual test; 254 (55.5%) did so when the child was six to ten years of age.

Conclusion: The current study showed poor awareness levels about pediatric eye diseases among parents, mainly regarding cataracts and glaucoma. On the other hand, parents demonstrated a high level of engagement in visually assessing their children. The primary hindrance to conducting assessments was the absence of symptoms and signs or active complaints, leading to a lack of proactive seeking of visual evaluations.

## Introduction

The importance of children's eye health can not be overstated as it has significant implications for personal life, education, career, health, financial status, and overall satisfaction. Additionally, it has a considerable economic burden and impacts the productivity and development of society. Conditions such as strabismus, congenital glaucoma, amblyopia, and refractive errors can lead to permanent vision loss if not addressed early on [1]. Early intervention is crucial in preventing complications and maintaining healthy vision. The World Health Organization (WHO) recognizes the elimination and control of childhood blindness as a priority through the "VISION 2020: The Right to Sight" program. According to WHO, 75% of blindness, regardless of age, can be prevented through therapeutic and preventive interventions [2]. Globally, 500,000 children lose their sight each year [1]. In the Middle East, 4.1% of total blindness cases are attributed to childhood blindness [3]. Refractive errors are the leading cause of visual impairment in children, but they can be treated and prevented from progressing to blindness in the long term [4]. It is worth noting that refractive errors among school-age children are on the rise [5].

Amblyopia affects 1-4% of children and can be caused by factors such as visual deprivation, strabismus, high refractive error, or anisometropia [6]. Detecting these abnormalities in young children can be challenging, but early intervention is of utmost importance in protecting their future vision. Vision screenings, performed by primary care providers or trained professionals, including school nurses, optometrists, or technicians, should be conducted throughout childhood to identify these issues. Utilizing a combination of screening evaluations yields higher sensitivity compared to a single evaluation, especially when different methods are used [7]. Unfortunately, many children only visit an ophthalmologist for their first eye examination at the age of six to eight years [8], at which point significant amblyopia, often anisometropic amblyopia, has already developed. This vision loss could have been prevented if the condition had been detected and treated earlier with the use of glasses. Therefore, it is crucial to emphasize the routine assessment of vision in all children, with special attention given to those with disabilities. Children with developmental delays may experience delays in identifying ocular diseases, impeding successful treatment. Early childhood vision screenings can reduce the risk of vision loss at the age of seven by over 50% [9]. This study aims to assess parental awareness of pediatric eye diseases, identifying gaps to develop targeted education for improved eye health in children.

## Materials and methods

A cross-sectional analytical study was conducted from August 2023 to December 2023. The targeted study population encompasses parents residing in Hail and Al-Qassim, Saudi Arabia. The inclusion criteria mandate participation by all parents in these regions who were willing to engage in the study. To ensure a representative sample, the study employed the simple random sampling technique, with a calculated sample size of 385 parents, determined by a 95% confidence interval. However, the final sample size was 618 after data collection. Data were collected through two pre-designed and pre-validated questionnaires formulated in prior studies conducted in Riyadh, Saudi Arabia [2], and Madinah, Saudi Arabia [10]. These questionnaires were amalgamated, redundant questions were removed, and some questions were reformatted to create a unified online questionnaire.

The distribution of the questionnaire was facilitated through a self-administered Google Form shared via WhatsApp, Snapchat, and X (formerly known as Twitter). This distribution method included the questionnaire, research objectives, and study rationale. The questionnaire underwent translation from Arabic to English and back for linguistic validation. To ensure the questionnaire's validity, a pilot study was conducted, and data from this preliminary phase were excluded from the main study. This meticulous approach aimed to enhance the reliability and robustness of the data collected. The questionnaire commenced with obtaining informed consent, ensuring the confidentiality of the data. It comprised 42 questions divided into four sections. The first section focused on gathering socio-demographic information, including the number of children and the presence of any eye diseases. The second section, consisting of five items, aimed to assess knowledge about eye care. The third section was designed to evaluate knowledge regarding common eye diseases in children, and the final section explored the practices of parents concerning children's eye care. The study ensures ethical considerations by obtaining informed consent from participants, maintaining data confidentiality, and adhering to research ethics standards. The Research Ethics Committee (REC) at the University of Hail reviewed and approved the study protocol under the reference number H-2023-438, as of December 18, 2023.

Data analysis

The data were collected, reviewed, and then fed to SPSS v. 21 (IBM Corp., Armonk, NY). All statistical methods used were two-tailed with an alpha level of 0.05 considering significance if the p-value is less than or equal to 0.05. Overall knowledge and awareness levels regarding pediatric eye problems were assessed by summing up discrete scores for different correct awareness items. The overall awareness score was categorized as a poor level if the parents' score was less than 50% of the overall score and a good level of knowledge was considered if the parents' score was 50-75% and excellent if the score was 75% or more. Descriptive analysis was done by prescribing frequency distribution and percentage for study variables including parents' data, and eye diseases and problems among children. Also, the parents' knowledge and awareness about pediatric eye diseases, practices, and attitudes were tabulated while overall awareness level and their source of information were graphed. Cross-tabulation for showing factors associated with parents' knowledge about pediatric eye problems was carried out with Pearson chi-square test for significance and exact probability test if there were small frequency distributions.

## Results

A total of 618 eligible parents were included, 429 (69.4%) from Al-Qassim and 189 (30.6%) from Hail. The parents' ages ranged from 20 to 59 years with a mean age of 35.4 ± 11.5 years. A total of 510 (82.5%) respondents were women; 569 (92.1%) were married and 608 (98.4%) were Saudi. As for education, 406 (65.7%) were university graduates, and 141 (22.8%) had secondary education. A total of 307 (49.7%) participants were non-healthcare workers, 278 (45%) were not working and only 31 (5%) were healthcare workers. A monthly income of less than 5000 Saudi riyals (SR) was reported among 208 (33.7%) parents and 188 (30.4%) had monthly income of 5000-10000 SR while 41 (6.6%) reported more than 20000 SR per month. Only 19 (3.1%) parents reported working in a field related to eye healthcare (Table 1).

**Table 1:** Socio-demographic characteristics of the parents included in our study SR: Saudi riyals

Socio-demographics	No	n (%)
Region		
Al-Qassim	429	69.4%
Hail	189	30.6%
Age in years		
20-30	67	10.8%
31-40	251	40.6%
41-50	247	40.0%
> 50	53	8.6%
Gender		
Male	108	17.5%
Female	510	82.5%
Marital status		
Married	569	92.1%
Divorced	31	5.0%
Widow	18	2.9%
Nationality		
Saudi	608	98.4%
Non-Saudi	10	1.6%
Educational level		
Below secondary	37	6.0%
Secondary	141	22.8%
University graduate	406	65.7%
Postgraduate	34	5.5%
Work field		
Not working	278	45.0%
Student	2	0.3%
Non-healthcare worker	307	49.7%
Healthcare worker	31	5.0%
Monthly income		
< 5000 SR*	208	33.7%
5000-10000 SR	188	30.4%
11000-20000 SR	181	29.3%
> 20000 SR	41	6.6%
Do you work in a field related to eye healthcare?		
Yes	19	3.1%
No	599	96.9%

Table 2 shows the eye diseases and problems among children of our cohort. A total of 190 (31.1%) parents had two children, 161 (26.4%) had three children, and 142 (23.3%) had only one child below the age of 15 years. A total of 289 (46.8%) parents reported having a child with an existing eye problem, namely, refractive errors among 183 (63.3%), amblyopia among 32 (11.1%), squinting among 34 (11.8%), and others. A total of 322 parents (95.2%) reported that their child wears glasses. A total of 237 (95.2%) of children with visual impairments attend school. Among non-attendants, the most reported reasons were parents not knowing where to send their child and/or children for schooling (50%), child and/ or children being teased by peers (41.7%), and being scared that their child and /or children would not be able to cope (8.3%).

**Table 2:** Reported eye diseases and problems among children of the parents in our study

Children eye problems	No	n (%)
If yes, the number of children		
1	142	23.3%
2	190	31.1%
3	161	26.4%
4	76	12.5%
5+	41	6.7%
Does your child have an existing eye problem?		
Yes	289	46.8%
No	329	53.2%
If yes, mention the problem (n=289)		
Refractive errors	183	63.3%
Amblyopia	32	11.1%
Squint	34	11.8%
Allergy	13	4.5%
Keratoconus	2	0.7%
Dryness	7	2.4%
Others	18	6.2%
Do any of your children wear glasses?		
Yes	322	52.1%
No	296	47.9%
Regarding the visually impaired child, do they attend school?		
Yes	237	95.2%
No	12	4.8%
Reasons for not attending (n=12)		
Don’t know where to send child and/or children for schooling	6	50.0%
Child and/ or children will be teased by peers	5	41.7%
Scared that the child and /or children would not be able to cope	1	8.3%

Table 3 shows the levels of parents' knowledge and awareness about pediatric eye diseases in our study. Regarding amblyopia, 42.7% of the parents correctly defined the disease, 33.2% correctly reported refractive error as the cause of the disease, 31.6% reported patching the strong eye as the treatment method, 79.9% knew that treatment led to better outcomes, and 51.8% knew that amblyopia leads to decreased visual acuity. Considering cataracts, only 12.6% correctly defined the disease, 31.7% knew it could affect children, 23.5% reported that having white pupils is a sign in the child, 21.2% knew that cataracts could lead to permanent blindness in children; however, 55.2% knew that surgery is the treatment of cataracts. 

**Table 3:** Parents' knowledge and awareness about pediatric eye diseases in our study (Part I)

Parents' Knowledge	No	n (%)
What is the definition of amblyopia?		
Decreased vision in one or both eye	264	42.7%
Degeneration of optic nerve	75	12.1%
Decrease night vision	3	0.5%
Misalignment of both eyes	69	11.2%
I don’t know	207	33.5%
What are the causes of amblyopia?		
Refractive error	205	33.2%
Strabismus	114	18.4%
Cataract	32	5.2%
TV and smart devices	240	38.8%
Hereditary	116	18.8%
Fever in infancy	37	6.0%
I don’t know	222	35.9%
What is the treatment of amblyopia?		
Patching the strong eye	195	31.6%
Glasses	129	20.9%
Eye muscle exercise	55	8.9%
Surgery and laser	41	6.6%
No need for treatment	4	0.6%
I don’t know	194	31.4%
Regarding treatment outcomes of amblyopia, does early treatment lead to better outcomes?		
Yes	494	79.9%
No	12	1.9%
I don’t know	112	18.1%
What are the complications of amblyopia?		
Decreased visual acuity	320	51.8%
Blindness	33	5.3%
Disability	7	1.1%
School failure	39	6.3%
Impaired quality of life	39	6.3%
I don’t know	180	29.1%
What is a cataract?		
A lens changes where the lens becomes opaque	78	12.6%
A white membrane growing over the eye	276	44.7%
A white spot in the eye	79	12.8%
I don’t know	185	29.9%
Do cataracts affect children?		
Yes	196	31.7%
No	43	7.0%
I don’t know	379	61.3%
How would you know if your child has cataracts?		
Would have white pupil	145	23.5%
Would have red eyes	15	2.4%
Would have pain in the eye	20	3.2%
Would have reduced vision	80	12.9%
I don’t know	358	57.9%
Can cataracts lead to permanent blindness in children?		
Yes	131	21.2%
No	52	8.4%
I don’t know	435	70.4%
What is the treatment for cataracts?		
Surgery	341	55.2%
Medications	37	6.0%
No treatment for cataracts is known	5	0.8%
I don’t know	235	38.0%
When surgery should be done?		
After birth	41	6.6%
Later on	186	30.1%
I don't know	391	63.3%

Table 4 shows parents' knowledge and awareness about pediatric eye diseases. A total of 19.3% correctly defined glaucoma, 17.2% knew that glaucoma can affect children, and 40.8% knew that it can cause blindness. Otherwise, 42.1% said that premature babies develop eye problems while 50.2% knew that a check-up by an ophthalmologist a few weeks after birth is the method to find out about potential eye problems in a premature child. Only 6.1% and 2.3% reported laser and cryotherapy as the treatment modalities for retinal diseases because of prematurity, respectively. Around 67.3% of the parents knew that children can develop blockages in tear drainage and 44.5% said that children should attend a routine eye exam annually. 

**Table 4:** Parent's knowledge and awareness about pediatric eye diseases in our study (Part II)

Parents' Knowledge, Continued	No	n (%)
What is glaucoma?		
Damage to the nerve of the eye due to high pressure	119	19.3%
High pressure in the eye	74	12.0%
An age-related process leading to a decrease in peripheral vision	118	19.1%
I don’t know	307	49.7%
Can glaucoma affect children?		
Yes	106	17.2%
No	58	9.4%
I don’t know	454	73.5%
Can glaucoma lead to blindness?		
Yes	252	40.8%
No	31	5.0%
I don’t know	335	54.2%
Can premature babies develop eye problems?		
Yes	260	42.1%
No	28	4.5%
I don’t know	330	53.4%
How to find out about eye problems in a premature child?		
Check-up by ophthalmologist a few weeks after birth	310	50.2%
By having a squint in the eyes	19	3.1%
By having discharge from the eyes	42	6.8%
I do not know	247	40.0%
What is the treatment for retinal diseases because of prematurity?		
Laser	38	6.1%
Cryotherapy	14	2.3%
Surgery	50	8.1%
No need for treatment	23	3.7%
I do not know	493	79.8%
Can children develop any blockage in tear drainage?		
Yes	416	67.3%
No	13	2.1%
I don’t know	189	30.6%
How frequently should a child have a routine eye exam?		
Every 1 year	275	44.5%
Every 2 years	41	6.6%
Every 5 years	25	4.0%
Only when the child reports a problem	142	23.0%
Not sure	135	21.8%

**Figure 1:**
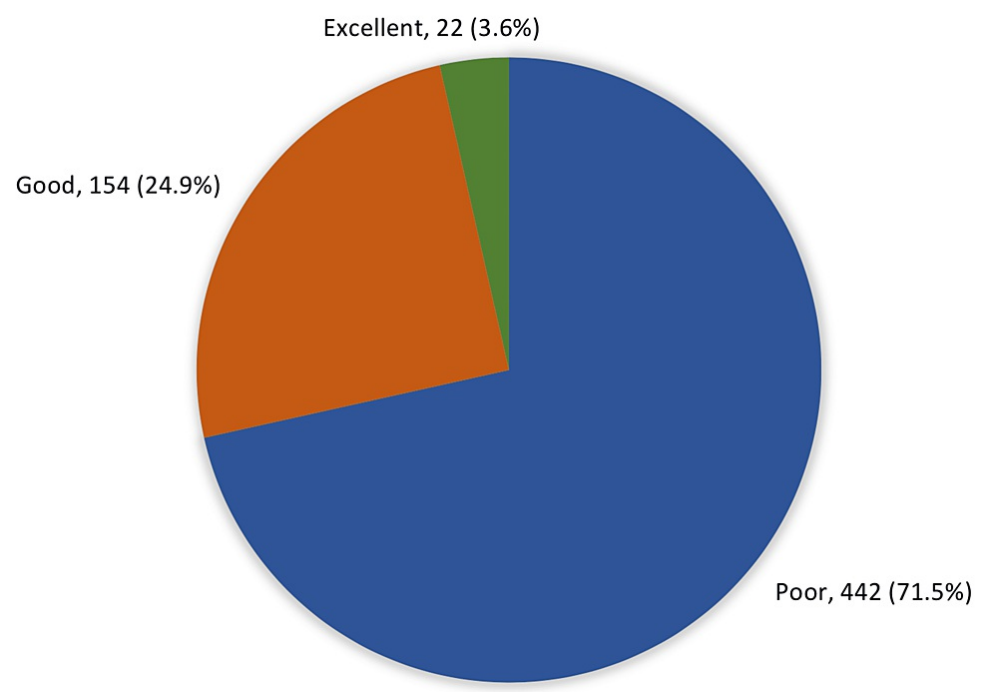
Overall parents' knowledge and awareness of pediatric eye diseases in our study

Figure 1 shows the overall levels of parents' knowledge and awareness of pediatric eye diseases. A total of 442 (71.5%) of the study parents had poor knowledge about their pediatric eye problems, 154 (24.9%) had good knowledge levels, and only 22 (3.6%) had excellent knowledge. 

**Figure 2:**
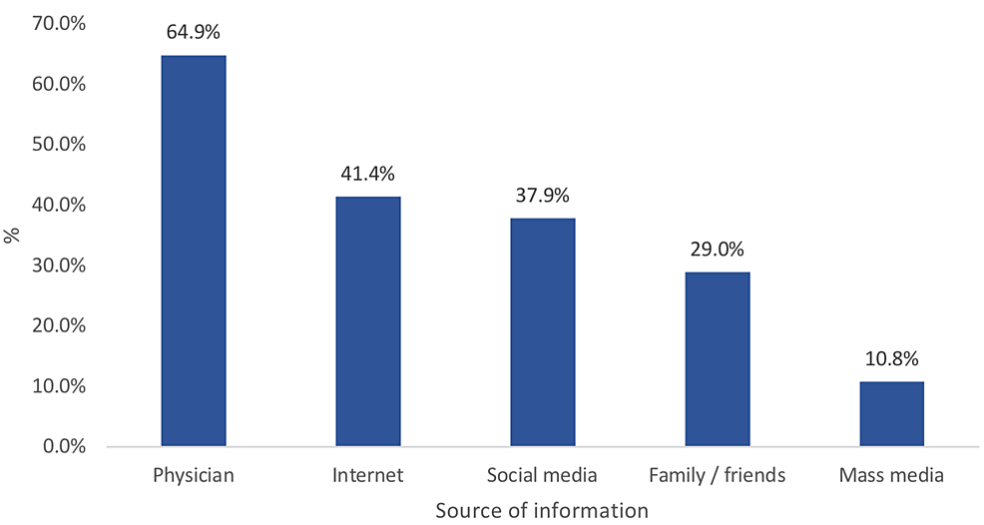
Sources of information regarding parents' knowledge and awareness of pediatric eye diseases in the Hail and Al-Qassim regions

Figure 2 shows the sources of parents' knowledge and awareness of pediatric eye diseases. The most reported sources were physicians (64.9%), the Internet (41.4%), social media (37.9%), family and friends (29%), and mass media (10.8%). 

Table 5 shows the parents' practices regarding their children's eye problems. A total of 458 (74.1%) of the parents in our study arranged for their children to undergo an eye or visual test; 254 (55.5%) did so when the child was six to ten years of age and 157 (34.3%) when their child was one to five years of age. Among those who did not, the most reported reasons included (i) they didn’t notice any signs that would prompt them to take their child to an eye doctor (58.8%), (ii) they felt their child was too young to have an eye test (26.3%), and (iii) they did not know how and/or where to arrange an appointment for an eye test (21.3%). As for observations that could prompt parents to take their child to an eye care practitioner, the most reported were child playing with toys at close range (53.2%), squinting of eyes (52.6%), complaints of double vision (51.9%), rubbing eyes repeatedly (49.8%), redness of the eyes (46.3%), excessive tearing (43%), and tilting the head to one side (30.7%). 

**Table 5:** Parents' practice regarding their children's eye problems in our study

Parents' Practice	No	n (%)
Has your child ever had any kind of eye or visual test?		
Yes	458	74.1%
No	160	25.9%
If the previous answer was yes, what was the age of the child when he/she was first taken for an eye exam? (n=458)		
< 1 year	47	10.3%
1-5 years	157	34.3%
6-10 years	254	55.5%
If the previous answer was no, what were the reasons? (n=160)		
I didn’t notice any signs that would prompt me to take him to an eye doctor	94	58.8%
I think my child is too young to have an eye test	42	26.3%
I don’t know how and/or where to arrange an appointment for an eye test	34	21.3%
I am worried about the cost of an eye test and glasses	24	15.0%
I am worried my child may be given glasses he/she doesn’t need	10	6.3%
Observations might prompt you to take your child to an eye-care practitioner		
Child playing with toys at close range	329	53.2%
Squinting of eyes	325	52.6%
Complaints of double vision	321	51.9%
Rubbing eye repeatedly	308	49.8%
Redness of the eye	286	46.3%
Excessive tearing	266	43.0%
Tilting the head to one side	190	30.7%

Table 6 shows parents' attitudes towards pediatric eye problems and their management. A total of 462 (74.8%) parents accepted the idea of their child wearing spectacles. Also, 484 (78.3%) would allow their child to undergo eye surgery if needed, but 14.2% would refuse due to fear of the outcome, and others would refuse due to other factors including fear of eye damage, cost, and cultural barriers. 

**Table 6:** Parents' attitude towards pediatric eye problems and management.

Parents' attitudes	No	n (%)
Do you accept the idea of your child wearing spectacles?		
Yes	462	74.8%
No	53	8.6%
Not sure	103	16.7%
Would you allow your child to undergo eye surgery if needed? If no, why?		
Yes	484	78.3%
No, due to fear of outcomes	88	14.2%
No, it would cause more damage to the eyes	7	1.1%
No, cost of the actual operation	15	2.4%
No, accessibility to services	5	0.8%
No, cultural and social barriers	19	3.1%

Table 7 shows the factors associated with parents' knowledge about pediatric eye problems. Around 6.9% of Hail region parents had excellent knowledge versus 2.1% of the parents from the Al-Qassim region (p=0.010). Also, 12.2% of parents with the highest income had excellent knowledge levels versus 2.4% of others with low income (p=0.003). Good-to-excellent knowledge about pediatric eye problems was measured among 32.2% of parents having a child with existing eye problems compared to 24.3% of others (p=0.020). Likewise, 32.6% of parents whose children underwent visual assessments had good-to-excellent knowledge compared to 13.7% of others (p=0.001). Also, higher knowledge was significantly detected among parents who received information from physicians and among those who accepted the idea of their child wearing spectacles. 

**Table 7:** Factors associated with parents' knowledge about pediatric eye problems P: Pearson X^2^ test; ^: Exact probability test *P <0.05 (significant)

Factors		Overall knowledge level						p-value
		Poor		Good		Excellent		
		No	n (%)	No	n (%)	No	n (%)	
Region	Al-Qassim	315	73.4%	105	24.5%	9	2.1%	0.010*
	Hail	127	67.2%	49	25.9%	13	6.9%	
Age in years	20-30	43	64.2%	19	28.4%	5	7.5%	0.435
	31-40	177	70.5%	66	26.3%	8	3.2%	
	41-50	180	72.9%	59	23.9%	8	3.2%	
	> 50	42	79.2%	10	18.9%	1	1.9%	
Gender	Male	77	71.3%	26	24.1%	5	4.6%	0.795
	Female	365	71.6%	128	25.1%	17	3.3%	
Educational level	Below secondary	30	81.1%	6	16.2%	1	2.7%	0.655
	Secondary	100	70.9%	37	26.2%	4	2.8%	
	University graduate	291	71.7%	99	24.4%	16	3.9%	
	Post-graduate	21	61.8%	12	35.3%	1	2.9%	
Work field	Not working	201	71.8%	70	25.0%	9	3.2%	0.095^
	Non-health care worker	225	73.3%	72	23.5%	10	3.3%	
	Health care worker	16	51.6%	12	38.7%	3	9.7%	
Monthly income	< 5000 SR	152	73.1%	51	24.5%	5	2.4%	0.003*
	5000-10000 SR	133	70.7%	49	26.1%	6	3.2%	
	11000-20000 SR	138	76.2%	37	20.4%	6	3.3%	
	> 20000 SR	19	46.3%	17	41.5%	5	12.2%	
Do you work in a field related to eye healthcare?	Yes	14	73.7%	3	15.8%	2	10.5%	0.188^
	No	428	71.5%	151	25.2%	20	3.3%	
Does your child have an existing eye problem?	Yes	193	66.8%	87	30.1%	9	3.1%	0.020*
	No	249	75.7%	67	20.4%	13	4.0%	
Has your child ever had any kind of eye or visual test?	Yes	304	66.4%	137	29.9%	17	3.7%	0.001*^
	No	138	86.3%	17	10.6%	5	3.1%	
Source of information regarding eye disease	Physician	259	64.6%	122	30.4%	20	5.0%	0.001*
	Social media	167	71.4%	57	24.4%	10	4.3%	
	Family/friends	130	72.6%	40	22.3%	9	5.0%	
	Internet	179	69.9%	65	25.4%	12	4.7%	
	Mass media	47	70.1%	16	23.9%	4	6.0%	
Do you accept the idea of your child wearing spectacles?	Yes	312	67.5%	128	27.7%	22	4.8%	0.001*^
	No	40	75.5%	13	24.5%	0	0.0%	
	Not sure	90	87.4%	13	12.6%	0	0.0%	
Would you allow your child to undergo eye surgery if needed? If not, why?	Yes, totally	336	69.4%	129	26.7%	19	3.9%	0.086^
	No, fear of outcomes	106	79.1%	25	18.7%	3	2.2%	

## Discussion

Annually, in the Middle East, approximately half a million children experience blindness, with 4.1% of all blindness cases affecting children [1,3]. The World Health Organization states that 75% of eye disease cases, regardless of age, can be prevented through therapeutic and preventive measures [4]. Studies on children’s eye diseases have highlighted the significance of raising awareness among parents to combat the problems of vision in their children. However, in Saudi Arabia, limited research has focused on parents' awareness and attitudes toward pediatric eye conditions and vision loss. The current study aimed to assess parents' awareness regarding pediatric eye disease and its management.

The current study showed that refractive errors were the main eye problem reported by 183 (63.3%) parents; also, the incidence of amblyopia and strabismus was much lower than refractive errors. Similarly, a previous study in Jazan revealed that strabismus, refractive error, ocular trauma, eye infection, and keratoconus were the most common eye diseases reported among children [7]. Also, according to several case studies, refractive error is the most prevalent morbidity disorder among children in the eastern province of Saudi Arabia [8]. Also, the study revealed that the vast majority of children with visual impairments attend school. Among non-attendants, the most reported reasons were parents not knowing where to send their child and/or children for schooling, child and/ or children being teased by peers, and worry that the child and /or children would not be able to cope. Shin et al. emphasized that visual impairments are a significant predictor of academic performance [11]. However, there is limited evidence on how vision affects the academic performance of school children. An Australian, series of studies on various grades of elementary school children was conducted, which concluded that better visual information processing results in better academic performance [12-14]. Additionally, in Malaysia, Chen et al. conducted a study in 2011 that included 1,103 Grade 2 school students, supporting the idea that visual performance plays a crucial role in learning [15].

Regarding parents' awareness and knowledge about pediatric eye diseases, the current study showed awareness for some and frequent eye diseases including amblyopia, cataracts, and glaucoma. As for amblyopia, less than half of the parents correctly defined the disease, one-third of them correctly reported the refractive error as the cause of the disease, and reported patching the strong eye as the treatment method. Considering treatment, most of the parents knew that treatment led to better outcomes and about half of them knew that amblyopia leads to decreased visual acuity. 

Considering cataracts, a poor awareness regarding the disease's nature was reported but about one-third of the parents knew it could affect children, and only one-fourth of them reported that having white pupils is a sign in the child. Also, one-fifth of them know that cataracts can lead to permanent blindness in children. On the other hand, more than half of the parents reported surgery as the treatment of cataracts. 

The situation for glaucoma was not so different from cataracts as poor awareness about the disease's nature was observed and also about its rate in children. A much higher percentage stated that it could cause blindness. In total, the vast majority of the study parents had poor knowledge about their pediatric eye problems, and only one-fourth of them had good knowledge levels. Similar findings were reported in 2023, where the results showed that 72.8% of the parents had poor awareness of pediatric eye diseases, 24.5% had good awareness, and 2.8% had excellent awareness [16]. Also, the current findings were consistent with previous studies that assessed poor knowledge in 78.2% of parents [10], whereas another study reported poor knowledge among 91.9% of parents [1].

Regarding parents' practice, the current study showed that around three-fourths of the study population had their children undergo eye tests; of these, more than half of the children were six to ten years of age, and one-third were one to five years of age. Among those who did not, the most reported reasons included they didn’t notice any signs that would prompt them to take them to an eye doctor, additionally, they felt their child was too young to have an eye test, and that they don’t know how and/or where to arrange an appointment for an eye test. Considering observations might prompt parents to take their child to an eye care practitioner, the most reported were child playing with toys at close range, squinting of the eyes, complaints of double vision, rubbing eye repeatedly, redness of the eye, excessive tearing, and tilting the head to one side. This is consistent with the results of a similar study has found that the majority of the parents believed in the importance of eye examinations and screening for their children [17]. Another study revealed that 65% of children undergo eye examinations [18].

Physicians were found to be the highest source of knowledge (64.9%), followed by the internet and social media. This finding was consistent with other studies [19,20]. However, another study has demonstrated that social media was the highest source of knowledge regarding eye diseases [21]. This finding indicates the power of social media in enhancing public awareness about eye disease.

A few limitations can be detected in this study. Responses were collected through an online survey that was not designed to be epidemiologically representative of a particular population; it contained a relatively higher number of females than males and more parents from Al-Qassim than Hail. The difference between the participants' categories (gender, age, region) should be minimized to compare their results accurately. The prevalence of children’s eye diseases was assessed through self-reporting by parents; a more accurate method such as a clinical examination or medical report could be implemented. 

## Conclusions

In conclusion, in our study, we aimed to assess the level of awareness of parents regarding pediatric eye diseases. We found poor levels of awareness about pediatric eye diseases among parents, especially for cataracts and glaucoma. According to this study, physicians were the primary source of information about pediatric eye diseases, followed by social media and the Internet. This result of poor awareness highlights the importance of increasing efforts to enhance parents' awareness of pediatric eye diseases and visual assessments, aiming to prevent potential consequences for their children's academic performance and daily lives.
